# PRIMARY CARE PHYSICIAN PERSPECTIVES ON THE INVOLVEMENT OF FAMILY MEMBERS IN THE CARE OF PEOPLE WITH DEMENTIA

**DOI:** 10.1093/geroni/igae098.2847

**Published:** 2024-12-31

**Authors:** Alma Hernández de Jesús, Sharifa Brooks-Smith-Lowe, Melissa Filippi, Julie Wood, Leah Karliner, Soo Borson, Alissa Bernstein Sideman

**Affiliations:** University of California San Francisco, San Francisco, California, United States; University of California San Francisco, San Francisco, California, United States; American Academy of Family Physicians, Lawrence, Kansas, United States; American Academy of Family Physicians, Leawood, Kansas, United States; University of California San Francisco, San Francisco, California, United States; University of Southern California, Santa Ana, California, United States; University of California San Francisco, San Francisco, California, United States

## Abstract

Involving family is integral to the care of people with dementia. Family medicine’s comprehensive “whole person” approach is ideally suited to this care. We interviewed 18 family physicians across the US to identify ways they involved and communicated with family members throughout dementia diagnosis and care. Interviews were thematically coded using ATLAS.ti. Participants identified several areas in which strong, open, and continuous communication with family made a difference in the care they provided to their patients with dementia: (1) Facilitating early diagnosis. Physicians in our study described the importance of family members in identifying the presence of cognitive impairment, communicating their observations to the physician, and providing information helpful in diagnostic evaluation. They also emphasized the importance of thoughtful communication with the family around disclosure once a dementia diagnosis is made; (2) Goal setting and care planning. Family physicians placed high value on supporting the patient’s quality of life, including prolonging life at home, and supporting patient autonomy. This included discussions about goals of care and intervening in challenging family dynamics; (3) Providing relevant disease education, facilitating access to resources, and acting as an interlocutor with specialists; and (4) Helping the patient avoid preventable hospitalizations by sharing safety measures and teaching family about managing medications and comorbidities. Although family physicians are uniquely suited to care for people with dementia, they also identified challenges to optimal communication: family members can be reluctant to address dementia care problems, and insufficient time, resources, and compensation challenge clinicians to sustain that communication over time.

